# Detection of Tickborne Relapsing Fever Spirochete, Austin, Texas, USA

**DOI:** 10.3201/eid2411.172033

**Published:** 2018-11

**Authors:** Jack D. Bissett, Suzanne Ledet, Aparna Krishnavajhala, Brittany A. Armstrong, Anna Klioueva, Christopher Sexton, Adam Replogle, Martin E. Schriefer, Job E. Lopez

**Affiliations:** Seton Medical Center, Austin Texas, USA (J.D. Bissett, S. Ledet);; Baylor College of Medicine, Houston, Texas, USA (A. Krishnavajhala, B.A. Armstrong, J.E. Lopez);; Austin Public Health, Austin (A. Klioueva);; Centers for Disease Control and Prevention, Fort Collins, Colorado, USA (C. Sexton, A. Replogle, M.E. Schriefer)

**Keywords:** Borrelia turicatae, bacteria, spirochetes, Ornithodors turicata, ticks, relapsing fever, diagnosis, elapsing fever spirochete, borreliosis, vector-borne infections, tick-borne infections, zoonoses, Austin, Texas, United States

## Abstract

In March 2017, a patient became febrile within 4 days after visiting a rustic conference center in Austin, Texas, USA, where Austin Public Health suspected an outbreak of tickborne relapsing fever a month earlier. Evaluation of a patient blood smear and molecular diagnostic assays identified *Borrelia turicatae* as the causative agent. We could not gain access to the property to collect ticks. Thus, we focused efforts at a nearby public park, <1 mile from the suspected exposure site. We trapped *Ornithodoros turicata* ticks from 2 locations in the park, and laboratory evaluation resulted in cultivation of 3 *B. turicatae* isolates. Multilocus sequencing of 3 chromosomal loci (*flaB, rrs*, and *gyrB*) indicated that the isolates were identical to those of *B. turicatae* 91E135 (a tick isolate) and BTE5EL (a human isolate). We identified the endemicity of *O. turicata* ticks and likely emergence of *B. turicatae* in this city.

Globally, spirochetes that cause tickborne relapsing fever (TBRF) are neglected pathogens, and diagnosis of this disease is challenging because of its nonspecific manifestations. Signs and symptoms of TBRF include cyclic febrile episodes, nausea, and vomiting (*1*). The bacterium *Borrelia turicatae* is the primary known causative species of TBRF in low-elevation, arid regions in the southern United States, and a unique manifestation of this disease is neurologic symptoms that further complicate an accurate diagnosis (*2*). In Texas, most cases of infection with *B. turicatae* have been associated with cave explorers, outdoor enthusiasts, undocumented migrants, and military personnel (*2*,*3*). Currently, it is not mandatory to report a diagnosis of TBRF in Texas.

An additional complication with defining public health effects of infection with *B. turicatae* is the dynamics between the pathogen and its tick vector (*Ornithodoros turicata*). These ticks have a life span of 10 years and can endure starvation for >5 years. However, *B. turicatae* remains infectious in these ticks (*4*). Except for persons bitten in karst formations (topographies formed from dissolution of soluble rocks) that can contain swarms of *O. turicata* ticks (*2*), attached ticks are rarely seen because these ticks are rapid feeders and transmission of *B. turicatae* occurs within seconds of the tick bite (*5*,*6*). In addition, *B. turicatae* is maintained transovarially and tick larvae are difficult to see because of their small size (*6*). After feeding, ticks return to their cavity dwelling, which includes wood cracks, leaf litter, and small- and medium-size mammal nests and dens (*2*,*7*,*8*).

Little is known regarding the maintenance of *B. turicatae* in nature. Most laboratory isolates have resulted from feeding field-collected ticks on immunologically naive mice and culturing the spirochetes from infected murine blood (*9*). *B. turicatae* has also been cultured from the blood of sick domestic dogs (*9*,*10*) and a human (*11*). Furthermore, there is an absence of *B. turicatae* isolates from wild vertebrates, which further limits understanding the etiology of TBRF.

We report clinical manifestations of TBRF for a patient from Austin, Texas, USA. Using species-specific genetic and antigenic markers (*12*–*14*), we identified the etiologic agent as *B. turicatae*. Because access to the alleged exposure site was not available, we collected *O. turicata* ticks in a nearby public park. Collected ticks indicated the endemicity of the vector to Austin. These ticks were evaluated for infection by feeding them on immunologically naive mice. We report the transmission and isolation of TBRF spirochetes in culture medium. Partial sequencing of the flagellin B (*flaB*), 16S rRNA (*rrs*), and DNA gyrase B (*gyrB*) genes (total 2,398 bp) indicated probable emergence of *B. turicatae* in Austin, Texas.

## Materials and Methods

### The Patient

The patient was a 34-year-old previously healthy woman whose illness began on March 29, 2017, when she had a headache, myalgias, arthralgias, and malaise. On March 30, 2017, she traveled to California on a previously scheduled trip and was febrile. The patient had a temperature of 104°F that increased to 105°F, at which point she sought medical treatment at an urgent care clinic. Complete blood counts, and levels of electrolytes, blood urine nitrogen, creatinine, and liver enzymes were within references ranges. She was given intravenous fluids, discharged with a diagnosis of a viral illness, and given instructions for symptomatic treatment of this illness.

Over the next 2 days, the patient still had a high fever, which prompted her to return to the urgent care clinic. Given her ongoing signs and symptoms, she was referred to a local hospital emergency department in California where she underwent computed tomographic imaging of her brain and a lumbar puncture for cerebrospinal fluid analysis. Computed tomographic imaging of the brain showed no abnormalities. Analysis of cerebrospinal fluid also failed to demonstrate abnormal findings. It was again concluded that she likely had a viral infection and was discharged from the emergency department with instructions for symptomatic treatment.

On April 2, 2017, she reported a blotchy maculopapular rash that began on her extremities and spread to her trunk. The rash was nonpruritic, persisted for several days, then gradually faded away. The patient returned home to Austin, Texas, with a temperature of 104°F and continued to have a mild headache in conjunction with intermittent fever. She did not have nausea, vomiting, or diarrhea. Given her ongoing symptoms, on April 9, 2017 she sought an evaluation at an acute care hospital emergency department. At the emergency department assessment, a hematoxylin and eosin–stained peripheral thin blood smear was prepared for evaluation of bloodborne pathogens.

### Real-Time PCR Analysis

We performed a real-time PCR assay on DNA extracted from the spirochete-positive peripheral thin blood smear. We scraped 10% of the contents of the slide with a scalpel and placed the contents in a tube containing 200 μL of phosphate-buffered saline (GIBCO, Gaithersburg, MD, USA). We then extracted DNA by using a QIACube (QIAGEN, Valencia, CA, USA), a tissue protocol, and an elution of 100 μL. A total of 5 μL of the eluted DNA extract was used per 20-μL final volume reactions with primers and probes specific for the *B. turicatae* glycerophosphodiester phosphodiesterase (*glpQ*) gene (forward primer 5′-GCCTGTCAGAATGAAAAA-3′, reverse primer 5′-CACCTCTGTGAGCTATAATT-3′, and probe FAM-5′-TGAGTATGACAAACAAAAAACCACCA-3′-BHQ) and the *B. hermsii glpQ* gene (forward primer 5′-TCCTGTCAGGGCGAAAAAAT-3′, reverse primer 5′-GCTGGCACCTCTGTGAGCTAT-3′, and probe FAM-5′-AGTCAAAACCAAAAATCACCA-3′-BHQ). The PCR was performed as described (*14*). A no template (DNA) sample was used as a negative control, and DNA extracted from *B. turicatae* and *B. hermsii* cultures were used as positive controls.

### Immunoblotting

We also performed immunoblotting for relapsing fever group *Borrelia* spp. and *B. turicatae*, as described (*3*,*13*,*15*). We subjected protein lysates from 1 × 10^7^ *B. turicatae* and 1 µg of recombinant *Borrelia* immunogenic protein A (rBipA) to electrophoresis by using Mini PROTEAN TGX Precast Gels (Bio-Rad, Hercules, CA, USA) and transferred them onto Immobilon polyvinylidene difluoride membranes (Millipore, Billerica, MA, USA). rBipA was produced as a thioredoxin fusion protein to facilitate solubility and is ≈15 kDa larger than the native protein (*13*).

We sent a deidentified serum sample collected 50 days after infection to Baylor College of Medicine (Houston, TX, USA) for evaluation. This sample was diluted 1:200 in Tropix I-Block Protein-Based Blocking Reagent (ThermoFisher Scientific, Waltham, MA, USA), and polyvinylidene difluoride membranes were probed for 1 hour. Recombinant protein G conjugated to horseradish peroxidase (ThermoFisher Scientific) diluted 1:4,000 was used as the secondary molecule, and antibody reactivity was detected by chemiluminescence using the Amersham Enhanced Chemiluminescence ECL Western Blotting Detection Reagent (GE Healthcare, Little Chalfont, UK).

### Collection of *O. turicata* Ticks

Because access to the alleged exposure site was not available, we selected a field site in a public park near the suspected exposure site. We determined that the park was in Austin by using the Jurisdictions Web Map maintained by the Enterprise Geospatial Service Program of the City of Austin (http://www.austintexas.gov/department/gis-and-maps). Collection efforts were performed in July and November 2017. We placed CO_2_ tick traps with dry ice as bait in locations with promising *O. turicata* tick habitats, as described (*7*). As ticks emerged from leaf litter, we stored them in vials labeled according to collection site and date. We collected 20 nymphal ticks from the first location in July and November 2017. We identified the second location in November 2017 and collected 30 nymphs from this site.

### Tick Feedings and Isolation of Spirochetes

All animal studies were approved by the Institutional Animal Care and Use Committee at Baylor College of Medicine. The laboratory animal program follows standards and guidelines established by the Association for Assessment and Accreditation of Laboratory Animal Care and the National Institutes of Health Office of Laboratory Animal Welfare. Animal husbandry was provided by trained veterinary staff and animal care technicians.

We randomly selected 10 *O. turicata* nymphs from each collection vial and allowed them to feed on ICR mice obtained from the Institute of Cancer Research (Philadelphia, PA, USA) (*16*). Animals were sedated by inhalation of isoflurane (Henry Schein, Melville, NY, USA), and ticks were placed on the shaved abdomen of mice and allowed to feed to repletion. Upon completion of the blood meal, ticks were stored in TTP TubeSpin Bioreactor Tubes (MidSci, St. Louis, MO, USA) and housed at 25°C and a relative humidity of 85%.

We examined the 3 mice for spirochete infection for 10 days by nicking the tip of the tail and expressing a drop of blood onto a microscope slide. A coverslip was placed over the blood and examined at 20× magnification by using a CX33 Trinocular Dark Field Microscope (Olympus, Center Valley, PA, USA). When spirochetes were observed, we obtained a terminal blood sample from the sedated mouse by using cardiac puncture. We centrifuged ≈500 μL of blood at 5,000 × *g* for 10 min and then inoculated 50 μL of serum into 4 mL of modified Barbour–Stoenner–Kelly (mBSK) medium (*17*). Cultures were grown at 35°C in an atmosphere of 5% CO_2_ and examined for spirochetes every 4 days. When exponential growth was reached, we passaged cultures into two 50-mL culture tubes containing fresh mBSK medium for isolation of genomic DNA and generation of stocks (stored in glycerol). For DNA isolation, we produced spirochete pellets by centrifuging the 50-mL culture tubes at 8,000 × *g* for 20 min and extracted genomic DNA as described (*18*). We designated the 3 isolates from the mice as BRP1, BRP1a, and BRP2.

### Plasmid Analysis and Genetic Typing of Relapsing Fever Spirochete Isolates

We performed reverse-field electrophoresis to resolve plasmid content, as described (*9*). In addition to BRP1, BRP1a, and BRP2, we compared genomic DNA plasmid profiles with the 91E135 isolate, which originated in Crockett County, Texas (*9*). Total DNA was used from the 91E135 isolate passaged 5 times and from the Austin isolates passaged 2 times after initial isolation from mice.

We subjected 500 ng of genomic DNA from each isolate to electrophoresis at 100 V for 15 min and at 80 V for ≈40 hours by using a PPI-200 Programmable Power Inverter (MJ Research, Inc., Waltham, MA, USA). Gels were stained with GelRed Nucleic Acid Stain (Phenix Research Products, Candler, NC, USA) according to the manufacturer’s instructions.

We performed multilocus sequencing for relapsing fever *Borrelia* *flaB*, *rrs*, and *gyrB* genes. Amplicons were generated by using specific primer sets (Table). For the *rrs* gene, we used primers UniB and FD3 to generate the amplicon, and the remaining internal *rrs* gene primers were used for sequencing. PCR conditions were an initial incubation at 94°C for 2 min, followed by 35 cycles at 94°C for 30 s, annealing at 55°C for 30 s, and an extension at 72°C for 3 min. After the last cycle, an extension was performed at 72°C for 5 min. We analyzed PCR products by agarose gel electrophoresis to confirm the expected molecular size. Each amplicon was sequenced by Lone Star Laboratories (Houston, TX, USA) by using specific primers (Table). Chromatograms were analyzed, and poor sequences were trimmed by using Vector NTI software (Life Technologies, Grand Island, NY, USA). We performed a BLAST (https://blast.ncbi.nlm.nih.gov/Blast.cgi) search with assembled contiguous DNA segments (contigs) to speciate isolates.

**Table:** PCR primers used in multilocus sequence analysis of tickborne relapsing fever spirochete, Austin, Texas, USA*

Gene locus and primer	Primer sequence, 5′ → 3′
*rrs*	
UniB†	TACAAGGAGGTGATCCAGC
FD3†	AGAGTTTGATCCTGGCTTAG
16s (–)‡	TAGAAGTTCGCCTTCGCCTCTG
16s (+)‡	TACAGGTGCTGCATGGTTGTCG
Rec 4‡	ATGCTAGAAACTGCATGA
P6 Rev	TTTACAGCGTAGACTACCAG
P8 For	AAACGATGCACACTTGGTGT
P10 Rev	ACATAAGGGCCATGATGATT
*flaB*	
flaLL§	ACATATTCAGATGCAGACAGAGGT
flaRL§	GCAATCATAGCCATTGCAGATTGT
*gyrB*	
gyrB 5′+3§	GCTGATGCTGATGTTGATGG
gyrB 3′§	GGCTCTTGAAACAATAACAGACATCGC

*flaB, flagellin B; For, forward; *gyrB*, gyrase B; Rev, reverse; *rrs*, 16S rRNA. †Reported by Le Fleche et al. (*19*). ‡Reported by Porcella et al. (*20*). §Reported by Barbour et al. (*21*).

## Results

### Initial Diagnosis of Relapsing Fever Borreliosis

The patient was afebrile on initial presentation to the emergency department, but a fever quickly developed (temperature 103.3°F). Her health deteriorated, and she became tachycardic (heart rate 125 beats/min) and mildly hypotensive (blood pressure 92/38 mm Hg). An initial complete blood count showed a leukocyte count of 6,700 cells/mm^3^, a hemoglobin level of 11.1 g/dL, and a platelet count of 145,000/mm^3^. Levels of liver enzymes, bilirubin, blood urea nitrogen, and creatinine were within reference limits.

At the hospital visit, the patient reported a travel history to Cancun, Mexico, during February 14–20, 2017, but she had no illness during or upon return from that trip. Also, she had no extensive travel history outside Austin, during February–March 29, 2017, when her illness began. However, during March 24–26, the patient spent a weekend at a rustic conference center in Austin where attendees from throughout the United States converged. Austin Public Health had previously investigated the conference center as the exposure site for an outbreak of TBRF the previous month, but tick trapping efforts were unsuccessful.

**Figure 1:**
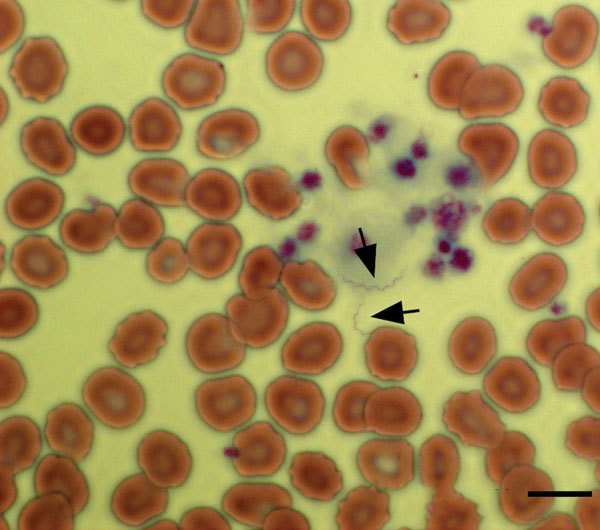
Giemsa-stained peripheral blood smear of a 34-year-old woman with tickborne relapsing fever, Austin, Texas, USA, showing 2 spirochetes (arrows). Scale bar indicates 20 μm.

During the conference, the patient noticed several insect bites on her legs. However, she did not report seeing ticks. Given her travel history to Cancun, the emergency department physician suspected malaria and requested a peripheral blood smear. Instead of malaria parasites, spirochetes were visualized (Figure 1) and a diagnosis of relapsing fever was made. Treatment with doxycycline was initiated on the evening of hospital admission, and her fever resolved within 24 hours. Modest leukopenia (leukocyte count 3,300 cells/mm^3^) and thrombocytopenia (71,000 platelets/mm^3^) then developed. Her other signs and symptoms rapidly resolved over the next 3 days, and she was discharged on April 12, 2017.

### Diagnosis of Exposure to *B. turicatae*

Because blood was not collected during the initial hospitalization of the patient to identify the infectious agent, we retrospectively implemented 2 molecular approaches to identify the causative agent. First, we extracted DNA from a portion of a blood smear and performed a real-time PCR with primers and probe specific for *B. turicatae glpQ* gene. This assay detected *B. turicatae* but did not detect *B. hermsii*. For this real-time PCR, the amplicon was 67 bp, and given the small size, we did not evaluate the sequence or submit it to GenBank. 

The average cycle threshold of the assay was 28.81, and use of primers and probe specific for *B. hermsii* showed negative results. A no template control also showed negative results.

**Figure 2:**
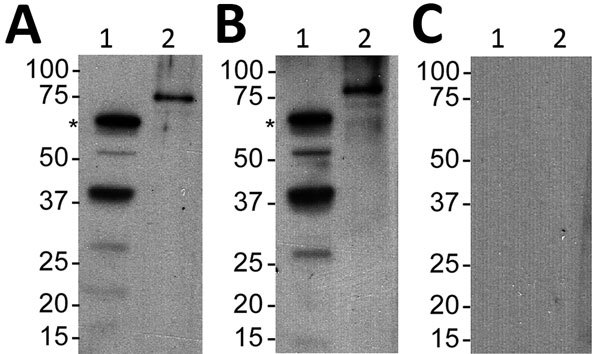
Serologic responses to *Borrelia turicatae* protein lysates and rBipA in 34-year-old woman with tickborne relapsing fever, detected by immunoblotting, Austin, Texas, USA. A) Serum sample from the case-patient; B) positive control serum sample from another case-patient; C) negative control sample. Lane 1, *B. turicatae*; lane 2, rBipA. Values on the left are in kilobases. Asterisks (*) indicate the size of native BipA. rBipA, recombinant *Borrelia* immunogenic protein A.

Second, we assessed a serum sample against *B. turicatae* protein lysates and rBipA. Results also indicated that the patient was infected with *B. turicatae* (Figure 2). Immunoblotting detected reactivity to 7 protein bands in the *B. turicatae* protein lysate and to rBipA (Figure 2, panel A). A positive control serum sample was used from a previous case report (*15*), and the negative control human serum sample indicated no serologic cross-reactivity (Figure 2, panels B, C).

### Evaluation of Infected *O. turicata* Ticks from a Public Park

**Figure 3:**
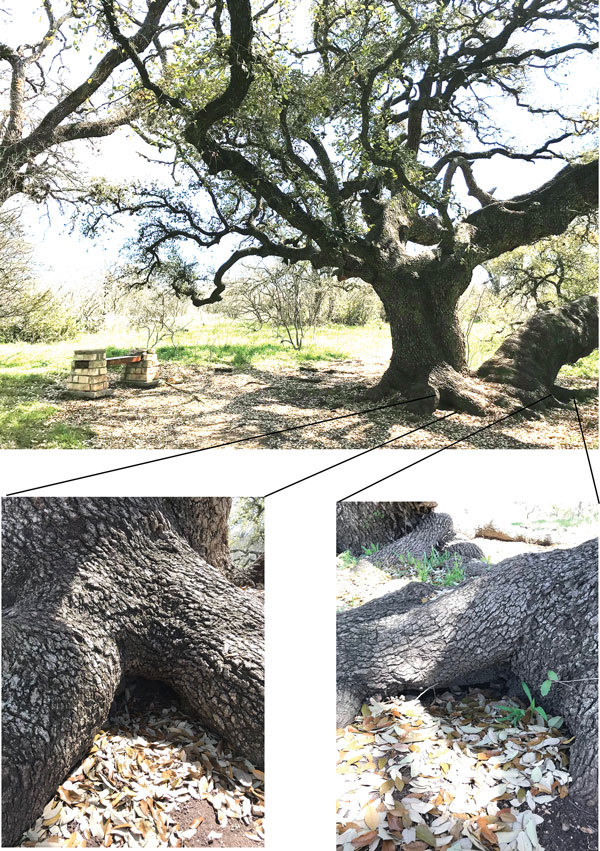
Location of collection sites for *Ornithodors turicata* ticks, Austin Texas, USA. Two rodent dens (insets) were located at the base of an oak tree. Carbon dioxide traps were placed at the openings until ticks emerged. *Borrelia turicatae* isolates BRP1 and BRP1a originated from ticks that were collected from the den shown at bottom left, and isolate BRP2 originated from the den shown at the bottom right.

We collected *O. turicata* ticks in a public park near the rustic conference center from the base of a tree that had 2 dens containing rodent waste, which suggested rodent activity in the area (Figure 3). Ticks emerged from leaf litter ≈20 min after we placed CO_2_ traps, and we collected ticks ≈30 cm from a cavity opening. Within 5 days after feeding ticks from each location on naive mice, spirochetes were visualized by microscopy in the blood. After we inoculated mBSK medium with a serum sample, spirochetes propagated. Once spirochetes entered the late logarithmic growth phase, we passaged them, which confirmed maintenance of these isolates by vitro cultivation. The 3 isolates were designated BRP1, BRP1a, and BRP2.

### Sequence Analysis and Plasmid Diversity of BRP1, BRP1a, and BRP2 Isolates of *B. turicatae*

We performed multilocus sequencing to characterize the 3 spirochete isolates that originated in Austin. Sequences of 1,400, 566, and 432 bases were generated for the *flaB, rrs,* and *gyrB* genes, respectively (GenBank accession nos. MH503949–51, MH507599–601, and MH507602–04, respectively). Assessment of DNA sequences by BLAST analysis indicated 100% nucleotide identity to the 91E135 and BTE5EL isolates of *B. turicatae.* Isolates 91E135 and BTE5EL originated from field-collected ticks and a febrile soldier from Texas, respectively (*3*,*9*,*11*).

**Figure 4:**
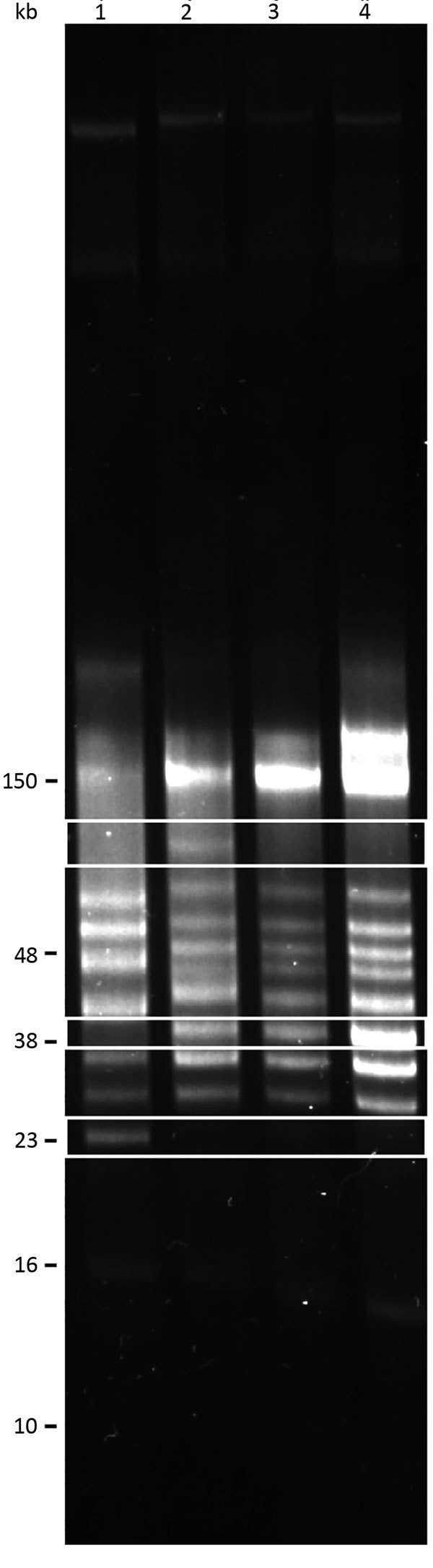
Reversed-field gel electrophoresis of *Borrelia turicatae* isolates collected from *Ornithodors turicata* ticks, Austin, Texas, USA (BRP1, BRP1a, and BRP2) and an isolate from previously field-collected ticks (91E135) (*9*). Lane 1, 91E135; lane 2, BRP1; lane 3, BRP1a; lane 4, BRP2. White boxes indicate a plasmid in BRP1 that is absent from the other strains (top); plasmids unique to BRP1, BRP1a, and BRP2 (middle); and a plasmid in 91E135 strain that is absent in isolates from Austin (bottom).

We performed reversed-field gel electrophoresis and identified variation in plasmid diversity between the BRP1, BRP1a, BRP2, and 91E135 isolates of *B. turicatae* (Figure 4). The 3 isolates from Austin contained an ≈40-kb linear plasmid that was absent from 91E135. In addition, BRP1 contained an ≈60-kb linear plasmid that was absent from 91E135, BRP1a, and BRP2.

## Discussion

We report TBRF in a patient from Austin, Texas. The patient had limited travel history outside Austin in the days before onset of signs and symptoms. Real-time PCR amplification of the *B. turicatae glpQ* gene and seroconversion for *B. turicatae* rBipA also helped identify the causative agent. Given that the suspected exposure site was previously investigated by Austin Public Health but ticks were not found, it was useful to collect *O. turicata* ticks near the site and assess their infectious status. Tick transmission feedings resulted in propagation of 3 isolates (BRP1, BRP1a, and BRP2), and results of multilocus sequencing of these isolates were identical to those of a human isolate and a tick isolate (*3*,*9*,*11*). Assessment of total genomic DNA indicated plasmid diversity between the 3 strains and the 91E135 isolate. Ticks that transmitted BRP1 and BRP1a were collected from the same den area at separate times during the year, which suggests circulation of >2 *B. turicatae* variants in the tick population.

Little effort has been invested in active surveillance of TBRF in Texas over the past 70 years (*22**,**23*), and more work is needed to understand the burden of this disease and maintenance of the spirochete in nature. Evidence indicates the endemicity of *O. turicata* ticks in San Antonio, Dallas, and Austin (*7*,*22**,**24*), the seventh, ninth, and eleventh most populous cities, respectively, in the United States. *O. turicata* ticks are considered an arthropod reservoir of *B. turicatae* because they have a 10-year life span and might endure years of starvation while retaining the ability to transmit *B. turicatae* (*25*). *O. turicata* ticks have also been collected in a variety of habitats, including caves, dens, rodent nests, and human dwellings (*2*,*7*,*9*). However, there is a paucity of information regarding mammals involved in maintaining *B. turicatae* in nature.

The life cycle of *B. turicatae* in the vertebrate host consists of periods when the bacterium is undetectable in the blood (*5*,*13*). Thus, indirect approaches are needed for improved surveillance. rBipA can support these studies because of the specificity of the protein to TBRF spirochetes (*12*,*13*). BipA homologs have not been identified in other pathogens, and previous studies indicated that serologic responses to the protein can discriminate between infections caused by Lyme disease and TBRF *Borrelia* species (*12*,*13*). Future efforts will focus on using rBipA as a novel diagnostic antigen for surveillance of *B. turicatae*.

TBRF is typically considered a disease of outdoor enthusiasts and impoverished persons living in primitive conditions (*1*–*3*,*11*,*15*,*26*). However, our study suggests emergence of *B. turicatae* in urban areas. The location where ticks were collected was in a densely populated region of the city. However, the maintenance of this pathogen in nature remains unclear. The elusive life cycle of *O. turicata* ticks also poses challenges in understanding the ecology of *B. turicatae.* Furthermore, given the nonspecific clinical manifestations of disease, the public health effect of *B. turicatae* remains vague. Our findings indicate that surveillance efforts should be increased in Austin, Texas, to evaluate emergence of TBRF.
